# The feasibility of a role for community health workers in integrated mental health care for perinatal depression: a qualitative study from Surabaya, Indonesia

**DOI:** 10.1186/s13033-018-0208-0

**Published:** 2018-05-31

**Authors:** Endang R. Surjaningrum, Harry Minas, Anthony F. Jorm, Ritsuko Kakuma

**Affiliations:** 1 Centre for Mental Health, Melbourne School of Population and Global Health, The University of Melbourne, Melbourne, Australia; 2 Faculty of Psychology, Airlangga University, Surabaya, Indonesia

**Keywords:** Community health workers, Integrated mental health, Perinatal depression, Health system framework, Indonesia, Primary health care

## Abstract

**Background:**

Indonesian maternal health policies state that community health workers (CHWs) are responsible for detection and referral of pregnant women and postpartum mothers who might suffer from mental health problems (task-sharing). The documents have been published for a while, however reports on the implementation are hardly found which possibly resulted from feasibility issue within the health system.

**Aims:**

To examine the feasibility of task-sharing in integrated mental health care to identify perinatal depression in Surabaya, Indonesia.

**Methods:**

Semi-structured interviews were conducted with 62 participants representing four stakeholder groups in primary health care: program managers from the health office and the community, health workers and CHWs, mental health specialists, and service users. Questions on the feasibility were supported by vignettes about perinatal depression. WHO’s health systems framework was applied to analyse the data using framework analysis.

**Results:**

Findings indicated the policy initiative is feasible to the district health system. A strong basis within the health system for task-sharing in maternal mental health rests on health leadership and governance that open an opportunity for training and supervision, financing, and intersectoral collaboration. The infrastructure and resources in the city provide potential for a continuity of care. Nevertheless, feasibility is challenged by gaps between policy and practices, inadequate support system in technologies and information system, assigning the workforce and strategies to be applied, and the lack of practical guidelines to guide the implementation.

**Conclusion:**

The health system and resources in Surabaya provide opportunities for task-sharing to detect and refer cases of perinatal depression in an integrated mental health care system. Participation of informal workforce might facilitate in closing the gap in the provision of information on perinatal mental health.

## Background

Community health workers (CHWs) in Indonesia are encouraged to be able to examine mental health problems experienced by pregnant women and postpartum mothers, which are stated in two policy documents: “the Guidance of Integrated Antenatal Care” [1] and manual for CHWs [2]. Nevertheless, despite policy and guidelines available, there is little data to indicate that this concept is being implemented.

**Fig. 1:**
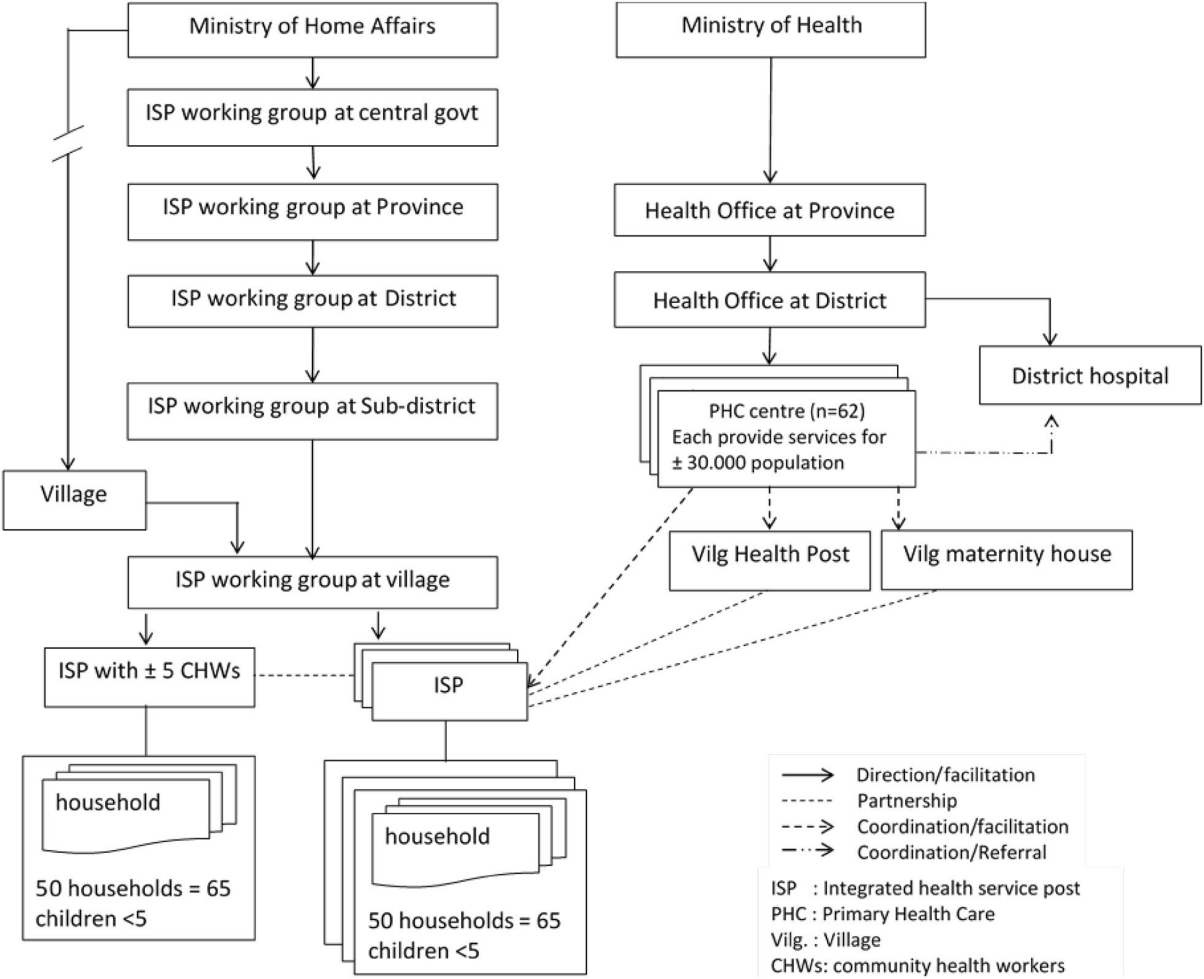
PHC system and networks

The prevalence of maternal mental health problems in Indonesia is under-recorded. Neither the national health survey conducted regularly every 5 years nor the annual Indonesia health profiles [3] present specific information regarding maternal mental health. Only a small number of epidemiologic studies on maternal mental health have been conducted, including one in Surabaya [4], that found the prevalence of perinatal depression was 22% [4, 5] (based on a cut-off point > 10 of Edinburgh Postnatal Depression Scale (EPDS), far higher than the reported global prevalence of 12% [6, 7]. Both the lack of information regarding implementation of policies on maternal mental health and the high prevalence of depression are reasons to initiate mental health care as part of maternal care in Surabaya, particularly identification of perinatal depression. Under the guidelines on integrated mental health in PHC [8], integrated mental health care could be developed. Within PHC system, there are integrated health service posts (ISPs) where CHWs work for maternal care in the community (see Fig. 1). A recent mental health policy accommodates the role of CHWs in mental health areas [9], even though not all administrative governments have implemented the decree. This policy provides an opportunity for task-sharing, i.e. CHWs to identify mental health problems in women during pregnancy and the postpartum period.

Task-sharing in general mental health care has been reported in other district in Indonesia [10] and other countries [11, 12]. This approach has also been applied for maternal mental health care [13–15]. In Surabaya, mental health care has been integrated into primary health care (PHC) centres, for several years [16–18], providing a basis for initiation of mental health care by CHWs [15, 19, 20].

While task-sharing in maternal mental health care has a legal policy foundation, annual reports indicated that it has neither been implemented in Surabaya nor known whether this is feasible and acceptable within this district health system [21–23]. To fill this knowledge gap, a comprehensive qualitative study was conducted to examine the feasibility and acceptability of task-sharing for perinatal depression, as well as the skills and competencies of CHWs to carry out the role. This article reports on a feasibility study based on perceptions of health system stakeholders.

### Role of CHWs within health system

The health system in Indonesia is administered in line with decentralization of the government system, such that services are decentralized to provincial and district governments under the Ministry of Home Affairs (MoHA) [24]. District governments operate health services provided through PHC centres called *puskesmas*, which typically reside in a sub-district. These centres supervise and support a wide network in the village level, including integrated health service posts (ISPs) known as *posyandu*, and village midwives (see Fig. 1).

An ISP involves intersectoral cooperation between the Ministry of Health and the MoHA at the village level [25], through a body called ‘ISP working group’ [26]. This working group coordinates with women’s agency of the MoHA called the family welfare movement (FWM) to run an ISP monthly activity [27] whose operationalization is managed by a PHC centre [28]. The FWM recruits CHWs who are volunteers from local community and allocate tasks for them. There are five main services of maternal and child health care at an ISP [28–30], therefore the FWM should ensure that there are at least five people to run an ISP. CHWs who specialize in this task are called CHWs^MCH^ in this article.

**Fig. 2:**
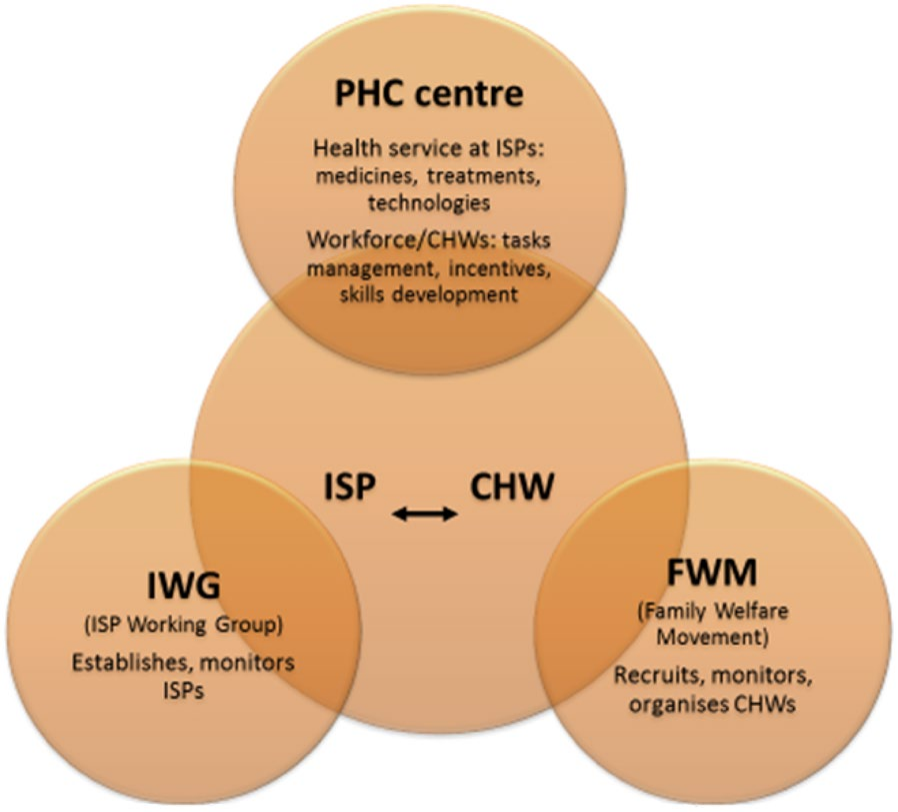
The relationship among ISP, CHWs, and PHC centre. PHC centre is health service providers at sub-district; an ISP is a community-based health care; CHWs are the community volunteers running an ISP; IWG is a village agency that establishes ISPs; FWM is women agency at the village that support ISPs

CHWs are responsible to assist health workers (e.g. village midwives) in maternal and child health care, nutrition advice and family planning during ISP activity; assist them in antenatal care such as organizing maternal classes; and undertake home visits for perinatal care [30]. Before taking the role, CHWs are trained in health-related areas by the health office, mainly in maternal and child health [30, 31], but also in other health areas. The relationship and roles of the PHC centre, FWM, and ISP working group in relation to the ISP and CHWs are presented in Fig. 2.

In certain circumstances, the role of ISPs extends to other health areas or areas other than health, such as social services and family welfare [30, 32]. When there are no people who have agreed to work voluntarily, a CHW^MCH^ may also be co-opted for this purpose, resulting in multiple roles for one CHW [32] including population and civil administration-related services [30].

### Aim of the study

The current study aimed to examine the feasibility of task-sharing of perinatal depression care in the health system in Surabaya. The aim was achieved through interviews to obtain perspectives of four types of stakeholders in the health system: (1) program managers, (2) health workers, including CHWs, (3) mental health specialists, and (4) service users.

## Methods

### Setting

The research took place at PHC centres in Surabaya that provide a psychological service, several ISPs managed by that PHC centre and a district hospital in Surabaya, and the District Health Office. There are 62 PHC centres in Surabaya and some of them provide psychological services. Three PHC centres were selected as the study sites: Centres A, B, and C, whereas Centre D was selected for a pilot study. They have different numbers of ISPs and Centre C was selected as it has the highest number of pregnant women, ISPs, and CHWs. ISPs were selected based on the centre’s advice.

### Participants and inclusion criteria

**Fig. 3:**
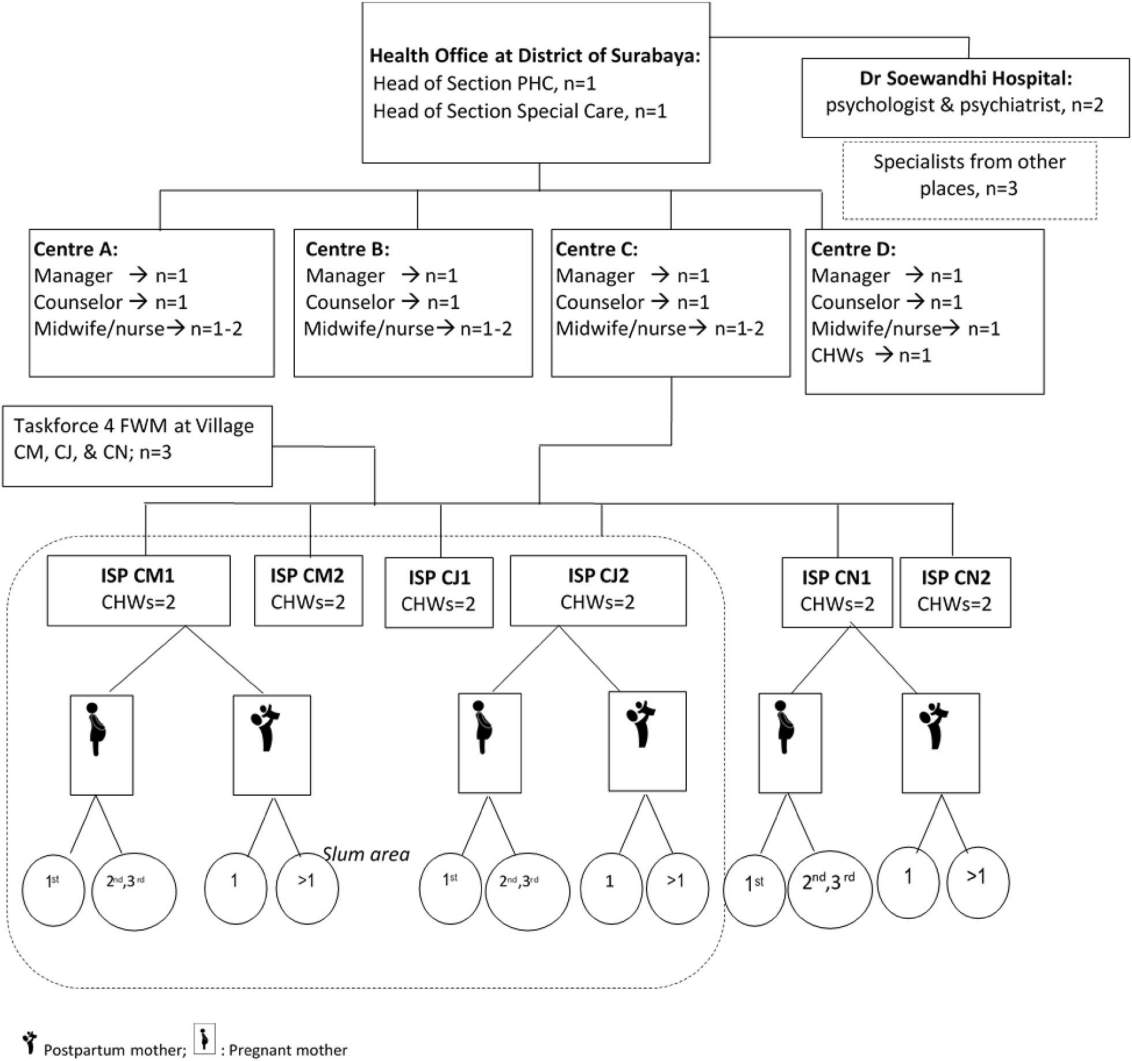
Research setting and the participants. *PHC* primary health care, *ISP* integrated health service post, *CHW* community health worker, *FWM* family welfare movement (*PKK*), *CM, CJ, and CN* sites for majority groups of population within Centre C coverage: CM for Madurese, CJ for Javanese, CN for non-slum area

Participants in the study were recruited from four groups of stakeholders: program managers, health workers, mental health specialists, and service users. Program managers consisted of two participants from the health office (district program managers) and three participants from three villages (community program managers). District program managers were the Head of the section of primary care service delivery that is in charge of maternal care and the head of the section of special health care that is responsible for mental health care. The three community program managers were members of an ISP working group from three villages which are responsible for organizing CHWs^MCH^ in the selected ISPs, viz. an ISP from Centre C with a high population of Madurese (ISP CM), one populated by Javanese (ISP CJ), and one from a non-slum area (ISP CN). Health workers comprised 12 formal health workers from three centres and 12 CHWs. Health workers were the centre managers, (mental health) counsellors, midwives, and nurses. CHWs were recruited from six ISPs, each of which typically has five CHWs and two of them were recruited: one leader (CHW-manager) and one member (CHW-member). Mental health specialists were a psychologist and a psychiatrist at the district hospital (Dr. Soewandhi Hospital). Two other psychologists and one psychiatrist were recruited from other places. Service users were 15 pregnant women and 13 one-year postpartum mothers (they will be called ‘women’ and/or ‘mothers’ interchangeably). Pregnant women were in their first pregnancy (primigravida) or subsequent pregnancy (multigravida) at any stage of pregnancy and had visited a health facility at least once. Postpartum mothers were mothers of a first child (primipara) or mothers of subsequent children (multipara). Two to three women were recruited from each ISP based on the percentage of pregnancy among women aged 15–49 [33]. The research setting and the participants are summarized in Fig. 3.

### Data collection

Semi-structured interviews were conducted individually by ES. The interviews consisted of questions on: (a) demographic information; (b) knowledge and attitude to mental health of mothers; (c) two vignettes about perinatal depression cases; and (d) the feasibility. Four different vignettes about perinatal depression cases were developed, two of which were presented to each participant according to their stakeholder group. The vignettes were presented before questions on whether participants have experienced a similar situation (for service users) or have dealt with similar clients (for service providers and community program managers), their perception of the importance of maternal mental health, and their views on the feasibility of task-sharing. Data collection was pilot tested in Centre D. All interviews were carried out in private settings, such as at home (for CHWs, pregnant women and postpartum mothers), or at their workplace (for health workers and program managers) and transcribed in Indonesian.

### Data analysis

Analysis of the data was carried out deductively with a framework analysis (FA) approach in the local language (Indonesian) using MS Word and the NVivo software program. Framework analysis has five key steps [34, 35]: (1) coding (indexing), (2) developing a working analytical framework, (3) applying the analytical framework, (4) charting data into the framework matrix, and (5) mapping and interpreting the data. WHO’s health systems framework was applied to direct the analysis. This framework suggests that a health system consists of six building blocks: health service delivery, leadership and governance, health workforce, health information system, medical products and technologies, and a health financing system [36]. Using MS Word, the researcher (ES) and an independent analyst, who is an Indonesian researcher holding a Masters degree from the University of Melbourne, read a set of transcripts from pilot interviews to identify emerging themes and to initiate the development of a thematic framework. The framework, which was presented in English, was then validated by the research team until a developing framework was agreed to be applied. The next step was indexing, which applied the framework to all the transcripts using NVivo 11. Frequent discussions with the whole research team took place throughout the data analysis phase, for which some transcripts were translated into English and analysis was evaluated by research team (RK), to ensure that interpretations were credible, valid, and shared.

### Ethics approval

Ethics approval for the study was obtained from the University of Melbourne (No. 1543833). A research permit was given by the Health Office of Surabaya. Informed consent was obtained from all participants prior to data collection.

## Results

### Participants

Recruitment of participants and interviews were carried out simultaneously from June to August 2015. In total, 62 participants from four groups of stakeholders were interviewed. The distribution of participants was as planned, but some adjustment was needed for the service users group. There was one pregnant woman from village CM who agreed to be interviewed but at the end refused to do so, whereas another pregnant woman from the same village agreed to participate in the study to enrich the voices of Madurese.

The demographic composition of the participants is as follow. Only four participants were male: three were specialists and the other one was a health worker. The ethnic background of the majority of participants was Javanese, with slightly more than ten percent being Madurese, and other ethnic groups comprising less than two percent. The age of participants was varied, but was above 25 years of age for all groups other than service users. All CHWs and community program managers were in their 40 s or above, had finished high school (junior and senior) but had no formal employment. Half of the service users were in their 20 s and only ten percent were under 20 years. More than three-quarters of these users had finished high school. Around 20% were primigravida, almost 70% had one or two children, and about ten percent had three or more children.

### Feasibility of extending CHWs’ role within the health system

**Fig. 4:**
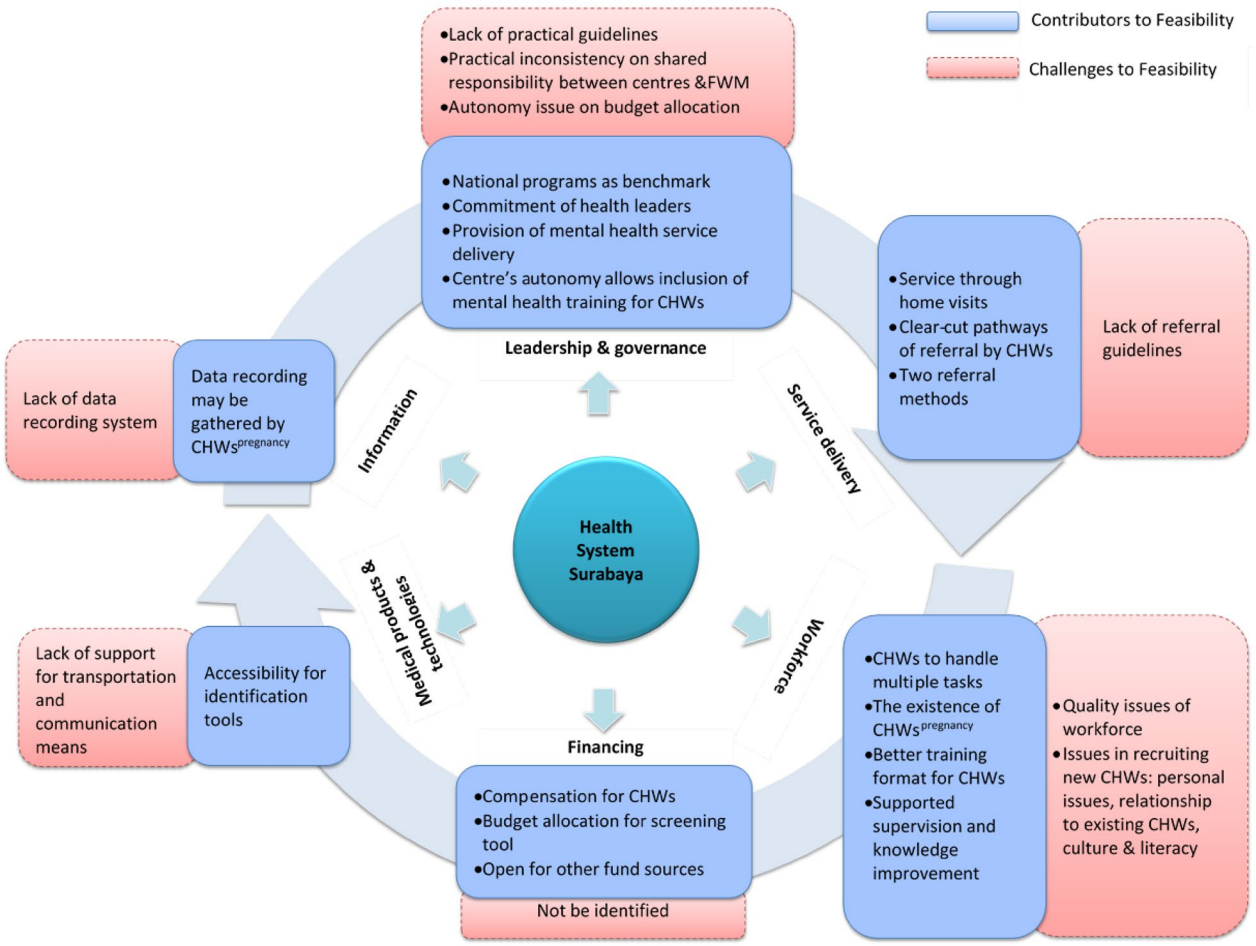
Factors contributing to feasibility of CHWs taking role in integrated mental health care in Surabaya

There were many shared views among stakeholders on factors that enable or hinder feasibility, as shown in Fig. 4.

#### Leadership and governance

Perceptions of feasibility based on leadership and governance factors were reported by program managers, a centre manager and a specialist. These participants described feasibility in these areas as being related to the availability of strategic and technical policies at national and district level, the intersectional collaboration between the general health service delivery sector and the special health care sector, and the autonomy of PHC centres to design context-specific programs.

#### The provision of national mental health policies

One factor that enables the feasibility of task-sharing in perinatal depression is the availability of two national policies on mental health. The first one is a national policy that shifted mental health care from hospital to community-based, called Community Mental Health Action Team (also known as *Tim Pelaksana Kesehatan Jiwa Masyarakat* or TPKJM*)*, adopted in 2002. The second policy is the current national strategic policy “*Indonesia bebas Pasung* (Indonesia Free from Shackles) *by 2015*”. A program manager stated that the latter program has become the pivotal point and timeframe for mental health actions, such as training on mental health detection to general practitioners and nurses in the centres, and out-reach activity to find mental health cases in the community. These policies do not refer to maternal mental health directly; however, they have brought about the establishment of mental health service units in several centres. The program manager for specialist care described how the policies have guided the formation of an out-reach team involving CHWs to find out people with mental health problems who live shackled. The significant number of cases found by the team generated attention to the importance of mental health issues and led to the establishment of mental health service units in several centres. She said:

*“Mental health is not a primary program, but an extended program. It has not got attention until during the outreach program*–*visiting patients at their original places*-*we found a lot of cases. Finally, the head office said ‘let’s develop your mental health unit’…. Because it should be free from pasung [shackle] by 2015, isn’t it?” (DPM 2)*

The manager added that two centres have established a mental health unit and another one is under preparation. Moreover, the community mental health action team policy led to the formation of a collaborative team across sectors to find and tackle mental health cases in the community based on their needs. Subsequently, the program manager explained that the team works regularly and is monitored directly by the Mayor.

*“…Tim Pelaksana Kesehatan Jiwa Masyarakat [Community Mental Health Action Team], TPKJM. Yes, we haven’t formed the structure, but I think… The team has not been structured, yet coordination among sectors has worked. Structurally it has not formed yet but the coordination has worked…. Usually each sector sends a letter to the Mayor and then the Office of Human Welfare would organize a meeting. These are from the Office of Social Affairs, of Health, of Housing…” (DPM 2)*

#### Health leader role

The involvement of the Mayor in coordinating the community mental health team was perceived as giving support and attention to mental health care in the city. A specialist reported how he was impressed by the Mayor:

“*The Mayor, indeed, believes in two principles, first there is no child in Surabaya who does not go to school, and second no sick people in Surabaya are neglected, including people with mental health problems….” (Sp 1)*

#### Coordination between sections in the district

Beside intersectoral collaboration conducted by the community mental health team, coordination between sections in the health office has worked well. The head of the section of general service delivery that organises maternal care felt sure that mental health care could be facilitated within maternal care. She said that integrated antenatal care is an obligation and the integrated care for mental health has been implemented by referring pregnant/postpartum mothers to the psychologist in the centre. She described:

*“It actually can be done, if the metal health [section] wants. Even though I am from maternal and child health I facilitate this…Those are compulsory, whether a mother is sick or not. An example of this is HIV assessment, it is compulsory…like that. When there is a complaint…yeah… it can be referred to the expert. You may have known that there are psychologists in several centres, so when there is a complaint or as a result of assessment by a GP or midwife, the patient would be referred to the psychologist… if there is no one in the centre, she may be referred to a psychologist at the closest centre.” (DPM 1)*

#### Policy on training of CHWs

Strategic policy at the district level on mental health training for CHWs is another factor to enabling feasibility. The training program for CHWs has been placed into the strategic planning for the following year, focusing on early detection and referral. The training was designed as an extension of the one provided for doctors and nurses. The program manager explained:

*“We have run training on early detection of mental health cases for health workers, and we plan to do so for CHWs this year….and in 2016 they will be trained in the basic knowledge of early detection and referral. That’s what we want to do.” (DPM2)*

The training at district level is more likely to be followed by centres because they have the autonomy to set up MCH-related training for CHWs in their area, allowing the inclusion of mental health in the program. Again, the manager emphasized:

*“It is the autonomy of the PHC to set up training materials for CHWs, not only about the health of children under five but anything that supports the health of those children.” (DPM 1)*

#### Challenges in implementation

In contrast to policy and planning that supports feasibility, several factors were viewed as challenges to feasibility. First, practical guidelines are required before task-sharing can be implemented in the community. A program manager made the point that even though a guideline has been regulated in the policy document, it may take several years until the guideline results in direct action. Second, there are practical problems with the shared responsibility between centres and the family welfare movement (FWM) in the village. Recruitment of CHWs is supposed to be a FWM task; however, a community program manager complained that in fact the PHC centre took over the task. Third, a centre manager was concerned about financial issues with the autonomy of a centre to arrange particular training for CHWs. Running a local program, which is centre specific is allowed, even though it is not part of a national or district priority, however the centre is responsible for the financial arrangement and the justification. The centre should find the money either from within its own or other funding sources and be able to justify the spending.

### Service delivery

#### Identification at an ISP is not feasible

This study was to assess the feasibility of task-sharing to be carried out by CHWs^MCH^ in their place of work, which is in an ISP. Nevertheless, stakeholders perceived that carrying out the task in an ISP is not feasible. Health workers stated that there are a lot of tasks to be accomplished by CHWs and that they are unable to also carry out task-sharing within the time available. In addition, the ISP was seen by mothers as a place for the health of children under five. Therefore, a lot of adults and children are around, resulting in a lack of sufficient space for privacy. A mother said:

*“Prefer during home visit. It is impossible to talk about my personal situation because the ISP is for children’s health…it’s better to visit home for that issue.” (Ppt A3)*

#### Service through home visit

In contrast to the ISP, carrying out task-sharing through a home visit was viewed as feasible. Home visits were expected, both culturally and officially. A CPM said that it is culturally accepted in the community to visit a mother who has just delivered a baby. She also stated that CHWs must carry out home visits when required, as part of a government program. A nurse emphasized that home visits have been conducted by CHWs^MCH^ to regularly monitor all aspects of the health of mothers and during these visits CHWs sometimes encountered women with mental health problems. For example, CHWs reported typical behaviours suggestive of a mental health problem, including isolation at home and missing check-ups during pregnancy.

Some CHWs viewed home visits as convenient, as they live close to users and the cost of transportation is minimal. Home visits also allow CHWs to gather more information from mothers, and enable them to approach women in more acceptable ways. A CHW explained:

*“I usually spend time to visit occasionally, during a spare time. So, it is not in a particular time, because it would be seen as a serious matter. Just pop in, have a chat, sharing as a neighbour friend.” (CHW J4)*

#### Referral

There are two types of referrals, internal and external, and CHWs could be involved in the former. Internal referrals are those that occur among professionals within a PHC centre. These procedures were illustrated by all program managers, some health workers, and by the CHWs. They explained that CHWs could refer depression cases in three formats: a verbal report directly or via telephone, a written report within a monthly report, and a written note in a communication book that delivers messages between CHWs and midwives at a PHC. The basic pathway for either format is from CHWs to village midwife to PHC centre midwife to counsellor at the centre (CHW → village midwife → centre midwife → counsellor). Sometimes a CHW and a village midwife go together to report a case, as one CHW described:

*“Two of us. Together. When we cannot handle it, we have a midwife coordinator and the coordinator will report to bu Nl [the centre manager]. If we cannot handle it, for example [because of] a psychological thing or need for a mental health consultation, we will go to Lk [the counsellor] ….” (Mw 3)*

CHWs might accompany mothers to the PHC centre when required. For other cases, the village or centre midwife and the counsellor come and visit the mother, either with or without a CHW. However, a centre manager was concerned about the lack of a referral book or note that provide details of the problem. Other health workers perceived that referral guidelines which describe the pathways and tasks of each professional are also needed.

External referrals send patients from the PHC to higher-level facilities. These referrals can occur when professionals at the centre cannot handle a health issue anymore, such as when a counsellor cannot handle a mental health case. A specialist and program manager at the health office explained that an external referral can only be made by a doctor in the centre and is directed to the district hospital. A specialist explained that even though a patient was being handled by a counsellor, the referral letter must be sent by a doctor.

### Workforce

The study participants raised numerous issues related to human resources. Concern was expressed about recruiting a CHW workforce of the quality required for work on perinatal depression. However, participants also perceived that the health system has training and supervision programs which could enhance skills and minimize such issues.

#### Availability and recruitment

The primary concern about workforce was the shortage of existing CHWs who qualify as suitable for task-sharing. Even for the general/current role, CHWs’ performance was often seen as being inadequate, due to their often being sick, being too old or their workload simply being too high. Program managers and health workers identified that most CHW-managers have multiple tasks related to their role in health assistance, i.e. in MCH, aged care, TB, dengue prevention, etc. A centre manager stated:

*“I have a lot of CHWs: CHWs*^*palliative*^*, I have a CHW*^*LKB*^ *who handles HIV and IMS [sexually transmitted disease], I have CHWs*^*TB*^*, leprosy and basically those for communicable diseases, and then CHWs*^*for*^ ^*nutrition*^ *called CFC [Community Feeding Centre] which are based at ISPs that handle malnourished*-*children. Sometimes only one person handles all of these because it is hard to recruit. But we think the person is able to handle all those roles.” (GP 3)*

Despite the recognition of high workload from multiple tasks, some health workers perceived CHWs as having the capability to handle those tasks. A program manager in the community suggested that CHWs could carry on several tasks in one go, referring to a strategy to manage tasks. This view underlined the feasibility in terms of human resources.

Having new recruits might be seen as an ideal solution to ease the overburdened CHWs and overcome the quality issue. However, there were some issues involved in getting new people. Firstly, because CHWs are lay community members volunteering their time to contribute to their community, other commitments such as work and domestic responsibilities were among the difficulties in recruiting new CHWs, as was described by some CHWs. A CPM listed social relationships as a second issue, when existing CHWs were seen as an obstacle to attracting and keeping new and younger CHWs. She illustrated this with the example of a potential woman who agreed to be recruited only if a particular CHW was not active in the taskforce anymore; and by another case in which a newly-recruited CHW stopped the role because she was treated badly by a current CHW. Additionally, cultural and demographic issues, such as ethnicity and literacy, came up when a CHW described a difficulty in engaging with women from a particular ethnic background because of their cultural beliefs and/or of low level of education.

Recently, a new group of CHWs, called CHWs^pregnancy^, was established to work for a PHC centre and the Family Welfare Movement (FWM) at the district level, which could also be seen as supportive of the feasibility of task-sharing and as an enrichment of the workforce. These particular CHWs have several tasks, including finding pregnant and postpartum women in the village and monitoring their health status through home visits, taking their pictures regularly to be documented, and making health record on monthly basis. The existence of CHWs^pregnancy^ and their tasks was described by a community manager:

*“Now we have what we call CHWs*^*pregnancy*^*. Here we have Wi, while Wa is from the next neighbourhood. One CHW would work for 2*-*3 neighbourhoods. They record pregnant women: how is their health status, the risks, including depression, and others. The CHWs monitor them until they give birth. To do so, the CHWs come to FWM representation at ‘dasawisma’ [smallest aggregate of neighbourhood] to collect the data on pregnant women in the area and then they visit the women at home. The community health centre also guides them. There are some in every village, for example this village has 6 CHWs*^*pregnancy*^*.” (CPM 1)*

#### Training and supervision

Training and supervision were perceived as other solutions for quality improvement that were available in the system. Counsellors from three centres reinforced the previous statement from a program manager that mental health training for CHWs and health workers has been carried out in the previous year. Unfortunately, follow-up of the training was challenged by staff rotation when the trained staff moved to a different centre, resulting in the program ceasing. While concerns were raised regarding the adequacy of training, the current strategic plan for training seems to be trying to address this issue. A counsellor said: *The health office has provided training for CHW*^*mental*^ ^*health*^*. But I think mere training is not enough; it needs to be continued with follow*-*up programs” (Cs 3)*. A district program manager expected that an already- established strategic plan for future training for CHWs^mental^ ^health^ from all centres would allow continuous training and sustainability of implementation.

*“That will be for next year. We will train them. For all centres. The previous one took only one day…therefore we will run the socialization so [the implementation] will not ‘come and go’ anymore.” (DPM 2)*

In addition, there is an opportunity for all centres to support the program and to provide supervision sessions for CHWs in the form of a monthly refresher program between health workers and CHWs. As well as supervision, the session is in fact also used for professional development when new and high-priority material needs to be introduced to the CHWs. Such a session could be used if mental health needed to become a topic area. Even better for feasibility is the fact that these regular sessions are financially supported by the health office.

### Information

Information systems that generate data about perinatal depression hardly exist, at either national, provincial or district level. Midwives and a district manager clarified that data on pregnancy and childbirth and mental health together is available, but there is no option to include information about mental health during pregnancy or across age. A CHW explained that a special case would be reported in a descriptive note within the regular maternal and child health recording sheet. Health workers suggested several potential ways of producing data on perinatal depression, basically through home visits or approaching pregnant and postpartum mothers directly and recording the information in a specific form. A nurse strongly suggested that the psychology unit could develop a form that could be completed by CHWs^pregnancy^:

*“…[CHWs* ^*pregnancy*^ *] are still working right now and they have to send a report, so it would be better if psychology can provide the sheet. But we may not find, I mean cases are not always found. So, when there are no cases they cannot just leave it blank, rather they still have to write a report, just write ‘nil’ for example.” (Nr 1)*

Another way was suggested by a CPM and CHWs-manager from three ISPs. They recommended that CHWs^pregnancy^ compile mental health information qualitatively, together with other health data for which they are responsible. Either way, the existence of CHWs^pregnancy^ was considered an opportunity for data collection on perinatal depression.

At present, a CHW^pregnancy^ reports data to the centre through village midwives and to the FWM at District level. While PHC centres use the data to determine service delivery to a mother, most CHWs and nurses did not know how the FWM uses this data.

*“There are CHWs for high risk of pregnancy; there are two of them: one is assigned by FWM at district level, and the other is assigned by the centre. In fact, they are similar in their role and responsibility… The one assigned by the centre will provide a report to us [village midwife] from which the report will be compiled into the MCH unit. The one assigned by FWM will send the report to the FWM at district level.” (Nr 1)*

### Financing

The financial feasibility of task-sharing is indicated by the availability of incentives for CHWs as compensation for taking the voluntary tasks, the budgeting policy to assign funding to support mental health screening during the pregnancy and 1-year postpartum period, and the open possibility for other funding sources. Currently, CHWs receive transport compensation for their role in each area they undertake (e.g. MCH, elderly, dengue) and, according to a district manager, they will also receive funding from the mental health program when it is set up. CHWs^pregnancy^ also receive incentives from the FWM or health office, depending on whom they work with. She emphasized that while the amount may be minimal, it shows the recognition of their roles. The other manager described the financial system assigned for PHCs through universal health coverage (the capitation fund) which could potentially be allocated to support depression identification in maternal care:

*“There is a solution to do so, using the capitation fund from universal health coverage. Here in Surabaya, which may be different from other districts, there is 60% from the operational budget to be addressed to services’ fee and 40% for others. The 40% will be divided into 30% for medicine and 10% for operational. One third of the 10% operational budget is targeted for health promotion programs which can be arranged for any required actions such as a goodie bag, leaflet, and so on. So, if mental health…, would possibly be printing a screening tool….” (DPM 1)*

A similar approach has been adopted to support supervision during the monthly refresher session. The manager added that funds from other sources are also accepted, such as from NGOs or the community. For example, there is an ISP in an exclusive residential area whose activities are fully funded independently by community members.

### Medical products and technologies

Medical products and technologies needed for depression identification by CHWs are basically related to the production of screening tools, and technologies for communication and transportation. CHWs currently detect mothers’ mental health in a common way using observation and then record the case as a note in the regular maternal and child health recording sheet.

*“They [CHWs] detected them in a common way: when a person isolates herself and never out from home and do not go for pregnant examination.” (Nr 2)*

There was a disagreement between two specialists on how the identification should be acted upon. One suggested two steps, starting with an interview and then following up with a scale. The other completely disagreed with CHWs identifying depression through an interview, as he believed that this requires a high level of knowledge and skill and therefore requires long-term training. He thought that a simple scale was preferable and he highlighted that tools for assessment of depression already exist and a simple one is quite easy to find.

*“For identification, it must use a tool that is internationally recognized, so using a depression rating scale is very simple…that is easy and the depression tools are not only one [type], from the simplest to the complex. It is so easy; indeed the tool to detect depression is easy so that we can teach CHWs. Identification using interview is more difficult, it needs a long time to educate CHWs.” (Sp 2)*

Means of communication and transportation are not necessary, as CHWs live close by the mothers and the village midwife is not far away. However, health workers and CHWs expressed concerns about the cost involved in taking mothers to the centre or if mothers live far away.

*“Because it is around the area, therefore transportation is not a problem. I would think twice if it is far away because I cannot ride a motorcycle and automatically I need to ask others to take me there.” (CHW J4)*

## Discussion

This study aimed to examine the feasibility of task-sharing in the identification of perinatal depression within the health system of the City of Surabaya, from the perspective of the health system’s stakeholders. Policy documents stated that CHWs can carry out this role [1, 2]. Results indicate that the proposed task is feasible to be implemented within the health system of Surabaya, from the perspectives of leadership and governance, home-based service delivery and internal referrals, training and supervision, financing, and technologies. Information systems and other areas need to be improved somewhat, including the ISP-based service, operational regulation, and workload of the volunteers.

Leadership and governance is a strong support for the involvement of CHWs in the identification of depression. The vision of both district government and the province as seen in mental health policy clearly indicates the potential for development of practice in this area. Indeed, mental health policy at the national level has had a significant development in the last two decades, and the lessons learnt can be useful in thinking about mental health policy for women and children. Perinatal mental health is an important component of mental health overall (with implications for both the mother and the child) and must be in one of the priorities within mental health. The recent development of mental health policies such as the mental health law and the law of persons with disability is progressive, which gives hope for the development of policies on women and children. This was seen when a new mental health law was approved by the house of representatives in 2014 (law number 18 year 2014), replacing the previous one that had been used for about five decades (the first mental health law was sanctioned in 1966). Not long after its release, another related law, the law of persons with disability, was authorized in 2016 (law number 8 year 2016) as a result of the ratification of the United Nation convention on the rights of persons with disabilities (UNCRPD). For a specific population, those affected by a disaster, the disaster mental health policy was developed in 2003 [37], while for those who have severe psychosocial disabilities living in physical restraint, an initiative from Aceh has been applied as a national program [38, 39]. These other laws/policies/initiatives can be used to advocate for high quality health and mental health care during the perinatal period.

Regarding the role CHWs in mental health care, the establishment of the TPKJM or community mental health action team in the district, whose performance is monitored by the Mayor, is another promising step for implementation of policy documents in task-sharing for perinatal depression. The fact that East Java is among the provinces that have established initiatives to implement TPKJM [40] is a good support for districts within the province such as Surabaya. The regulation is also reinforced by the policy on “Indonesia Free from *Pasung* (shackle)” launched in 2010, that was aimed to be achieved by 2014 (it has since been extended to 2019) [39]. These two national programs and the commitment of the leaders to them are evidence of a good foundation of health leadership and governance, and are most likely to support task-sharing in the mental health area. In particular, policies with a timeline, such as the free from shackle policy, seem to be having a greater impact, because the government is putting in greater effort to meet the goal within the schedule. This phenomenon accentuates WHO’s suggestion that a policy maker should have a timeline in mind when developing a mental health policy [41].

The policy emphases that facilitate task-sharing were also strengthened by organisational management in the health office. The existence of mental health within the special care section provides open opportunities for the mobilization of more resources in the health system. This can be seen from its roles in: facilitating the establishment of centres with mental health units; in putting in place a strategic plan for mental health training, including the training of CHWs; assigning a source of funding for depression identification; and organizing multisectoral collaboration that could support resource management, including the application of integrated antenatal care. Not all these efforts are right now directed specifically to the mental health of mothers; however, there is potential within the section for commencing subsequent steps to realise the vision.

According to stakeholders, home visits are the possible answer for the service delivery model for CHWs in carrying out the task of depression identification. As a model of care, this approach is not a novel one within the national health system, particularly for CHWs^MCH^. Several documents openly regulate this responsibility and provide structured guidance on what and how to conduct home visits [2, 30], even though not specifically for mental health care. For example, home visits are directed for mothers whose children under five did not attend an ISP activity, and those of malnourished children, among others. The home visit approach is also used by CHWs^pregnancy^, many of whom are also CHW^MCH^ (mostly the managers). At a practical level, there is agreement among stakeholders that a home-based approach is the best option to overcome the space limitation issue, the difficulties of accessing primigravida, and time constraints which resulted from many services being provided during an ISP activity and the unsuitability of this schedule for working mothers. This approach allows for flexible scheduling, as has been stated by CHWs and users, and is consistent with findings from another study [42].

Service delivery should also be connected to infrastructure and resources in the health system to make it feasible for task-sharing. Resources could provide a wider opportunity to assure that users get continuity of care after being identified by CHWs, e.g. infrastructure for referrals. It means task-sharing is supported by relevant continuous care so that the care does not end with the CHWs or village midwives. Continuity of care could also be understood in terms of protecting the rights of users to get treatment. The complete resources available for mental health care are: (1) the provision of mental health care by a counsellor in a PHC centre, (2) the availability of two district hospitals that provide mental health services by both psychiatrists and psychologists, and (3) the provincial mental hospital that is located in the city. With these resources, community-based mental health care fits within the national health system. Lack of continuous care was a concern for women who were reluctant to disclose their feelings during a mental health assessment [43]. Findings from another study have suggested that mental health screening as part of integrated routine maternity care would be a possible intervention pathway [44] which would involve less stigma. Participants in that study emphasized the unease and feeling of shame from talking about their experience of depression with multiple professionals in a fragmented care system, something that is not required in integrated care. In addition, continuity of care is supported by the financing system of universal health coverage. The economic cost of perinatal depression is high for both individuals (mothers and the family) and the public sector [45], therefore the health coverage scheme needs to make service delivery and referral procedures easy and accessible. The connection with other health systems, such as the PHC and the social welfare system, is also needed for well-functioning mental health care.

The financial source and policy in financing both support task-sharing, congruent with the arguments related to service delivery and continuity of care. How the capitation budget could be allocated so that a specific amount could be used to establish a depression screening tool was clearly explained by a district manager. This financial policy would open several possibilities for further steps, for example to identify and validate a simple and locally-acceptable screening tool. Studies from a variety of settings suggest several possible perinatal depression screening tools that can be accessed worldwide and have good psychometric properties [46–48], however adaptation in the new context is required. Several studies in Indonesia have reported the use of some of these tools (e.g. the Edinburgh Postnatal Depression Scale) [4, 5, 49]), nevertheless there is a need to examine whether similar tools can be administered by CHWs. Specialists in this study also emphasized the use of simple tools and believed that CHWs are able to administer them. Health workers suggested using a symptom list which is simple to administer and quite similar to a pregnancy risk scale with which CHWs are familiar. Another method is using a structured interview, but this was debated among specialists, since it requires a high level of competence. Furthermore, a specialist strongly suggested not using the word ‘depression’ to avoid stigma, with the term ‘mood changes’ being preferable instead. This suggestion is in line with previous findings in which the experience of depression was expressed in many forms and terms by Javanese [50]. This means that an understanding of the personal and cultural terms should also be considered in choosing or adapting an identification tool. Possible bias in interpreting a woman’s mental health state resulting from unfamiliar terms requires attention, considering the level of education, language and ethnicity of both the CHWs and users.

In regard to human resources, the findings on workload, scarcity, and personal barriers are consistent with feasibility issues reported by previous studies [51, 52]. These issues may result from the role of CHWs as the frontline workforce for many governmental sectors, not only health and home affairs, but also others such as education and social services [32]. At least 12 roles for CHWs have been listed in health-related areas [30], not including others in civil services [53]. Management of CHWs by the FWM is supposed to enable organization and monitoring of the availability, distribution, and performance of CHWs. However, it seems that the FWM, even at the national level, does not have a strong bargaining position in the governance of a village when a new task for CHWs is released. In fact, the findings about social relationships as one recruitment issue suggest that the FWM is the agency that best understands the social boundaries and cultural life of the community and so is best placed to map human resources in the area. Several concerns should be addressed to improve workforce management and quality, such as regulation of skills and characteristics required, the need for a working contract that regulates the length of employment and a procedure for terminating the role, the training required, and a means of distributing tasks. Well-distributed tasks may prevent duplication, so that new and existing CHWs^pregnancy^, for instance, could monitor not only physical but also mental health efficiently. Even though CHWs are volunteers, having professional management of their tasks would maintain their participation sustainably.

There is promise for developing and improving CHWs’ skills and competencies in task-sharing. This could be achieved through the availability of mental health counsellors at centres and the health office, and mental health specialists at district hospital. Skills enhancement programs are an opportunity for quality assurance in service delivery. Components of mental health training for CHWs are found in several studies [10, 40] that could be a source to learn from, including those addressing perinatal mental health [54, 55]. Nevertheless, since task-sharing in maternal mental health care has not yet started, the specific skills and competencies, as well as training, that fit the local context need to be examined.

### Challenges and recommendations

The findings indicated three main challenges to feasibility. These are: (1) inconsistency between policy and practices, (2) an inadequate support system for data management and technologies, and (3) unsupported means for implementation. Gaps between policy and action are revealed from the shortcut practices in recruiting CHWs by the centre instead of by the FWM. Several approaches may alleviate these challenges, such us inviting all parties (FWM and PHC centres) to sit together and review the policies, or hearing about the best possible strategies for a collaboration process before a proposed program is released. However, this study did not explore this possibility further. The fact that there were gaps between written regulations and the reality generates a concern: even if stakeholders’ perceptions lead to the conclusion that the health system can feasibly accommodate task-sharing in integrated mental health, personal views on participation may be different. Buist, O’Mahen [56] reported a mixed attitude to the acceptability of perinatal depression detection among women and health providers. Therefore, it is necessary to understand the personal views of stakeholders about the acceptability of their involvement in task-sharing.

Other issues are logistical support and an information system on perinatal depression. While transportation is not a significant barrier, because the task is within walking distance, the findings imply a need to use telecommunication devices in doing the job. Lack of financial support for communication and transportation is an issue for task-sharing in another area [10] and a similar problem is anticipated by CHWs in this study. In addition, the lack of an adequate data reporting system on perinatal depression could be solved through the use of qualitative reports from CHWs to village midwives to the centre. In an annual report, the health office presents data on mental health cases other than maternal ones [23], suggesting an opportunity to do the same for maternal mental health data. This possibility is suggested by the existence a mental health qualitative report shown by a district program manager during an interview when describing how the data were collected. Moreover, a study on maternal mortality calculation suggested the important role of village midwives and local registers (volunteers) in gathering and reporting valid data [57]. The study highlights the opportunity to integrate mental health as a component of health data collected by CHWs^pregnancy^.

**Fig. 5:**
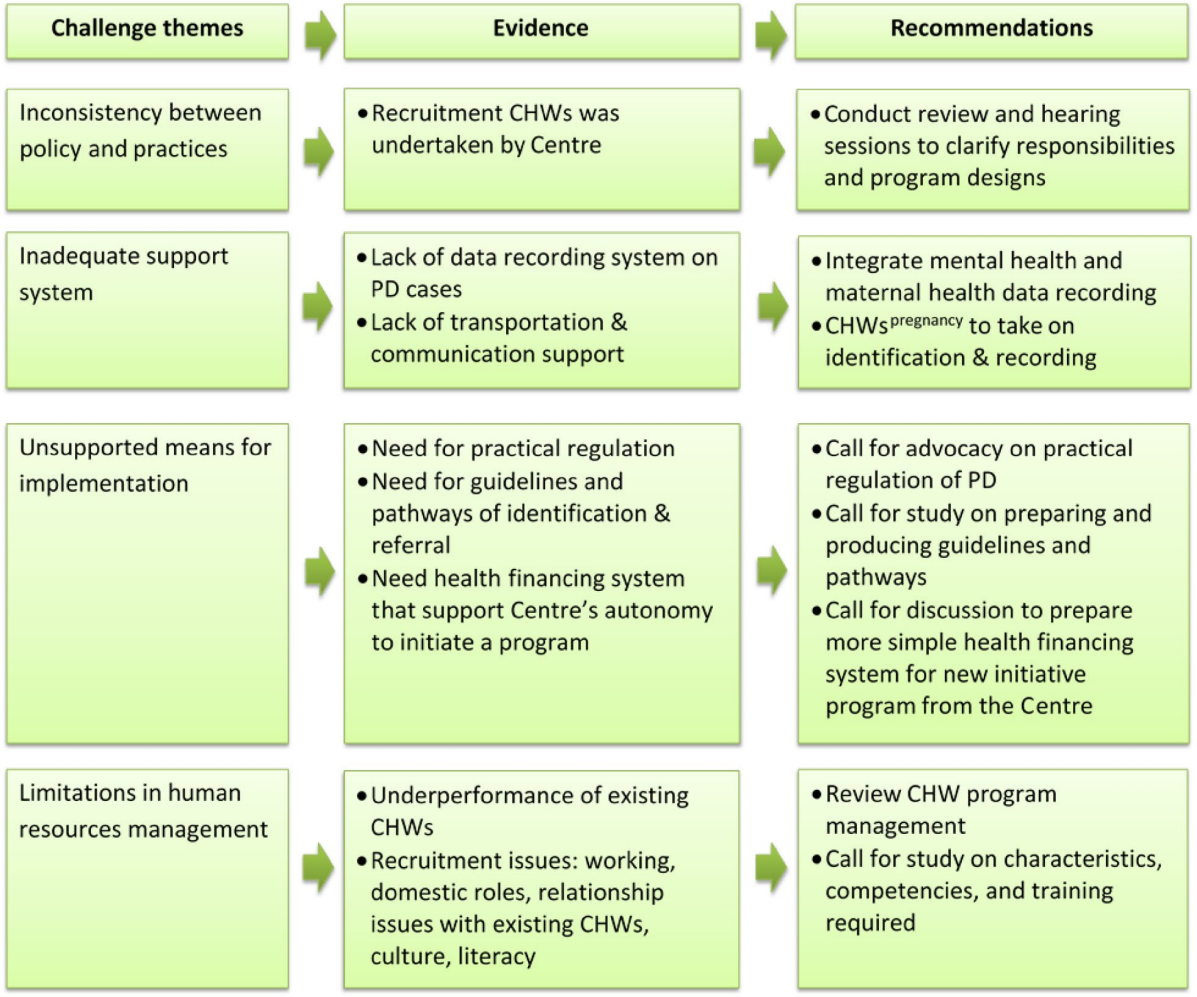
Challenges and recommendations of CHWs playing a role in task-sharing in mental heath care in Surabaya

Finally, the lack of practical regulation for task-sharing and the need for practical guidelines and pathways for identification and referral were anticipated as potential issues for implementing task-sharing. The health office should prepare staff in order to manage practices and roles of each actor. Another challenge for implementation is financial barriers faced by a centre if it has the will to initiate a program. The centre needs to allocate a budget or to find funding from other sources and justify it in a way that is acceptable within the health financing system. The procedure is perceived as a significant difficulty by centre managers. Figure 5 presents a summary of challenges and recommendations.

### Limitations

The current study has several limitations. First, the study was purely dependant on the perceptions of participants, which may not represent the mechanism and procedure in the health system. Using this strategy can produce unrealistically sound findings that do not fully identify challenges in the financial area, which is a typical obstacle in many reports. Second, the study was conducted in one district under decentralized governance, therefore extrapolation of the results to other contexts is limited. Third, the participants are only from health system tiers and do not extend more comprehensively to home affairs sectors. This strategy leaves several unanswered questions, such as the management of data by the FWM. Moreover, the number of areas sampled to represent the PHC centres was quite small compared to the total number of centres in Surabaya, even though it is quite large in the context of a qualitative study using individual interviews. This limitation has, however, been alleviated through the recruitment strategy for the research sites which took account of the socio-economic and cultural diversity of the city.

## Conclusion

It can be concluded from this study that the health system and resources in Surabaya are sufficient for the feasibility of task-sharing in integrated maternal mental health to detect perinatal depression. Most health system areas support or provide an opportunity for this concept, with there being a strong basis in governance and resources. The decentralized governance of the health system allows contextualization of a national policy. The role of CHWs also demonstrates their potential for filling the gap that exists in the data information system. Further studies are necessary before the idea can be prepared for implementation, including, but not limited to, exploring the acceptability of task-sharing and the characteristics of CHWs required for this purpose.

## Abbreviations

CHWs: community health workers
CHWs^MCH^: community health workers working for maternal and child health
CHWs^pregnancy^: community health workers working for pregnant mothers
CM, CJ, and CN: sites for majority population groups within Centre C, which CM for Madurese, CJ for Javanese, CN for non-slum area
EPDS: Edinburgh Postnatal Depression Scale
FWM: family welfare movement
GP: general practitioner
HIV: human immunodeficiency virus
ISPs: integrated health service posts
MCH: maternal and child health
MoHA: Ministry of Home Affairs
NGO: non-governmental organization
PHC: primary health care
TPKJM: community mental health action team
WHO: World Health Organisation
